# Another power of antibody-drug conjugates: immunomodulatory effect and clinical applications

**DOI:** 10.3389/fimmu.2025.1632705

**Published:** 2025-08-20

**Authors:** Ruotong Shi, Lin Jia, Zheng Lv, Jiuwei Cui

**Affiliations:** Oncology Department, Cancer Center, The First Hospital of Jilin University, Changchun, China

**Keywords:** antibody-drug conjugates, immune checkpoint inhibitors, tumour micro-environment, immunoregulation, tumour immunology

## Abstract

Antibody-drug conjugates (ADCs) enhance tumour immunogenicity through multidimensional immune modulation beyond targeted cytotoxicity. The immune remodelling of the tumour microenvironment (TME) suggests potential synergistic mechanisms with immune checkpoint inhibitors (ICIs): ICIs amplify antitumour immune responses by blocking inhibitory signals. Preclinical studies and preliminary clinical evidence demonstrate their synergistic efficacy; however, mechanistic synergy requires further experimental validation. Current challenges encompass the impact of heterogeneous TME on therapeutic outcomes and toxicity risks including interstitial lung disease. Advancing the translational potential of combination therapies necessitates optimised linker designs, development of immunostimulatory payloads, and establishment of precise biomarker frameworks. This review investigates the immunomodulatory mechanisms of ADCs, providing a theoretical foundation and novel directions for antitumour combination therapies and next-generation ADC development.

## 1 Introduction

ADCs, or antibody-drug conjugates, are a class of targeted-cytotoxic anticancer therapies with three major components: an antibody, a linker, and a payload (1). The payload of a classical antibody-drug conjugate is usually borne by a chemotherapeutic drug (cytotoxic drug), the antibody plays a targeting role, and the linker combines the two main components mentioned above to ensure that the drug exerts its efficacy only after it reaches the target tissue (2). The anti-tumour effects of ADCs are achieved through three main mechanisms: the target-specific cytotoxicity, the blockade of cell signal transduction pathways, and the immunologic regulation (3–5). ADCs are targeted, cytotoxic, and relatively long-term anti-neoplastic agents. Moreover, the long-term anti-tumour effect comes from its positive modulation of the anti-tumour immune effect, which enhances the immunogenicity of the tumour microenvironment (6, 7).

The regulation of anti-tumour immune activity by ADCs covers many aspects, the most critical being its regulation of various immune components in the tumour immune microenvironment (7, 8). The immunogenicity of the TME affects the response level of tumour tissues to anti-tumour drugs, and the more active the TME is, the better the prognosis of patients will be (9–11). A large number of studies have shown that ADCs can regulate the phenotypic differentiation of immune cells, the infiltration level, the secretion level of inflammatory factors, and the immunomemory formation in multiple dimensions. As a result, the immunogenicity of TME could be improved and the strength of anti-tumour immune response could be significantly increased (6, 12–15).

Considering the upward regulating effect of ADCs on the tumour immune microenvironment, some researchers have combined it with anti-tumour immunotherapy, intending to obtain better efficacy (14, 16–18). The anti-tumour effect of ICI(immune checkpoint inhibitor), depends on the target antigen expression level and the tumour microenvironment’s immunogenicity (19–22). The combination of ADCs and ICIs has been shown in clinical studies to provide patients with more significant benefits than either therapy alone (23–27).

Contemporary investigations into antibody-drug conjugates remain disproportionately centred on their tumour-killing efficacy, with scant attention accorded to their immunomodulatory functionalities. This review focuses on the modulating effect of ADCs on anti-tumour immunity. It aims to provide a more comprehensive and systematic account of the immunomodulatory effects of ADCs. And it also collate information on the relevant clinical trials of the antibody-drug conjugate and immune checkpoint inhibitor (ADC-ICI) therapy to provide theoretical support for the new therapy’s clinical application.

## 2 Immunomodulatory mechanisms of antibody-drug conjugates

**Figure 1:**
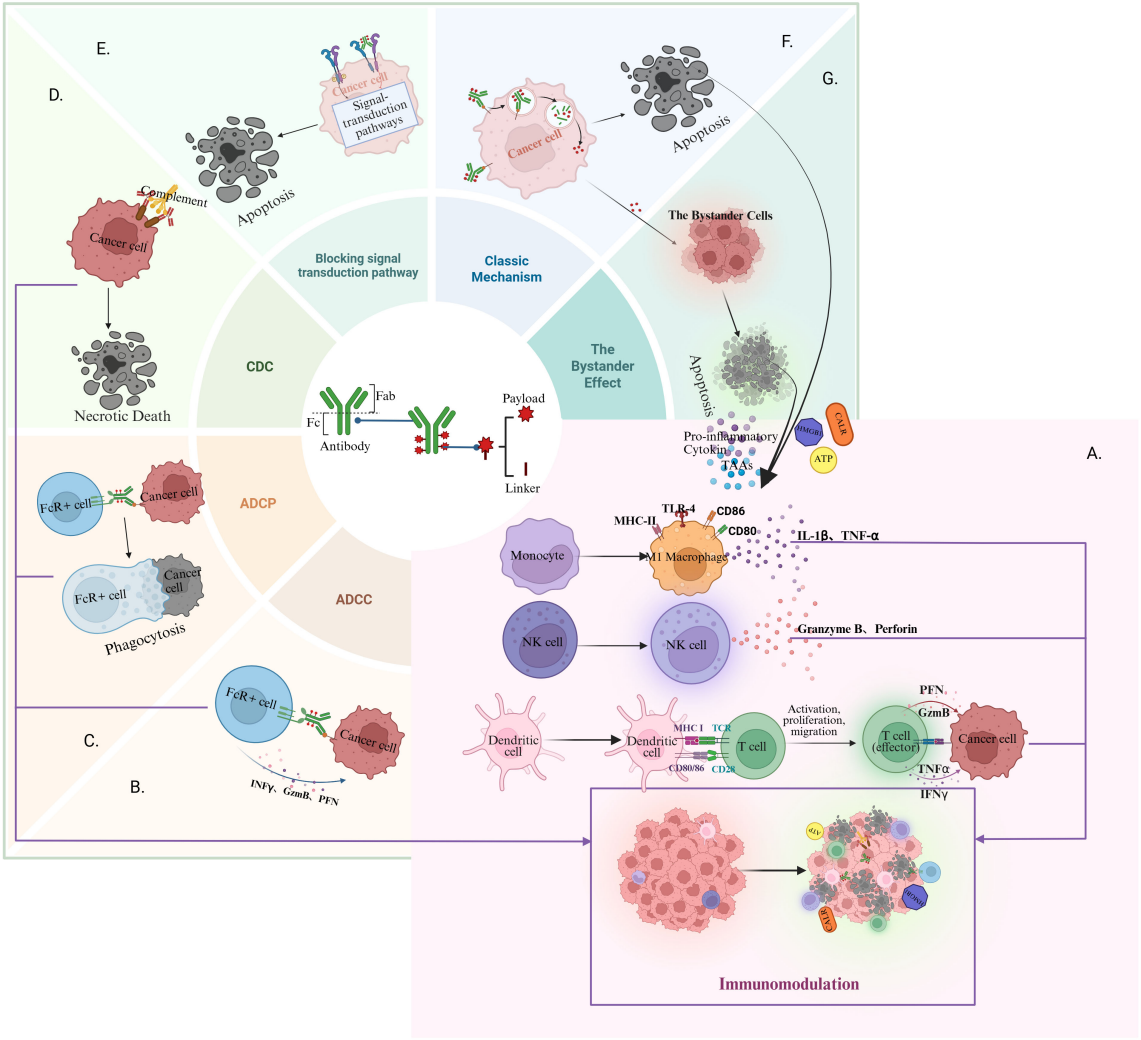
Key components and mechanisms of action of ADCs: **(A)** ADC enhances immune component activity within the TME; **(B)** ADC mediates tumour cell killing via the ADCC pathway; **(C)** ADC induces tumour cell elimination through the ADCP pathway; **(D)** ADC triggers the necrotic death of tumour cells via the CDC pathway; **(E)** ADC blocks cell signalling transduction to inhibit tumour growth; **(F)** ADC is recognised and internalised, and the cytotoxic payloads are released into the TME; **(G)** The released cytotoxic load can cause apoptosis of surrounding tumour cells via the bystander pathway. Created with Biorender.

Antibody-drug conjugates (ADCs) exert antitumour effects through a three-pronged mechanism: (1) Targeted cytotoxicity against antigen-expressing tumour cells; (2) Bystander effect-mediated elimination of adjacent malignant cells; and (3) Immunomodulatory remodelling ​of the tumour microenvironment (TME), enabling sustained therapeutic efficacy (4, 5). The immunoregulatory functions of ADCs primarily arise from payload-induced immunogenic cell death (ICD) and synergistic contributions of the antibody component (**Figure 1**).

### 2.1 Payload-mediated immunoregulation in ADCs

Cytotoxic payloads, including topoisomerase I inhibitors and monomethyl auristatin E (MMAE), ICD by disrupting DNA replication or microtubule dynamics. This process releases three key immunogenic signals: (1) Increase in damage-associated molecular patterns (DAMPs) such as extracellular adenosine triphosphate (ATP), high mobility group box 1 (HMGB1), and surface-exposed calreticulin (CALR), activating the anti-tumour immune effects; (2) Enhancement of cross-presentation of tumour-associated antigens (TAAs), which could prime T cell responses; and (3) High expression of pro-inflammatory cytokines like IFN-γ, which recruit and activate NK cells and T lymphocytes (6, 28–30). Brentuximab vedotin (BV), an anti-CD30-MMAE conjugate, exemplifies this mechanism by elevating IL-10 and IL-18 levels, thereby reprogramming the TME towards an immunostimulatory state (28). The ICD cascade establishes a self-amplifying loop that enhances immune-mediated tumour clearance and sustains DAMP release (14).

### 2.2 Antibody-dependent immune activation

The Fc domain of ADCs engages innate immunity through antibody-dependent cellular cytotoxicity (ADCC), phagocytosis (ADCP), and complement activation (CDC). These mechanisms enhance tumour lysis by natural killer cells(NK cells) and macrophages (31–33). Cleavable linkers (e.g., valine-citrulline) further amplify cytotoxicity by enabling payload diffusion to neighbouring cells, a phenomenon termed the “bystander effect.” This not only broadens tumour cell killing but also releases TAAs for cross-presentation, thereby bridging innate and adaptive immunity (34). High drug-to-antibody ratios (DARs), as exemplified by trastuzumab deruxtecan (T-DXd, DAR=8), maximise bystander activity, whereas non-cleavable linkers (e.g., in trastuzumab emtansine [T-DM1], DAR=3.5) restrict payload diffusion (35). T-DXd eradicates antigen-heterogeneous tumours via this mechanism while promoting dendritic cells (DCs) activation and durable immune memory (36).

### 2.3 ADC-driven remodelling of the tumour immune landscape

ADCs orchestrate multicellular immune activation within the TME: For dendritic cell activation, ADCs upregulate co-stimulatory molecules (CD80/CD86) and MHC-II on intratumoural DCs, enhancing antigen presentation (13, 37). Dolastatin-derived payloads (e.g., monomethyl auristatin E [MMAE]) further augment DC maturation, migration to lymph nodes, and T cell priming (30). Moreover, ADCs polarize tumour-associated macrophages (TAMs) towards pro-inflammatory M1 phenotypes by upregulating toll-like receptor 4 (TLR4) and suppressing scavenger receptor class A member 5 (SCARA5), thereby enhancing phagocytosis and IL-12 secretion (38). STING-targeting ADCs (e.g., αEGFR-172) synergize with DCs and NK cells via IFN-I signalling to amplify antitumour immunity (39). As well as NK cell engagement: ADCs activate NK cells via Fc-mediated ADCC (e.g., CD107a degranulation triggered by gemtuzumab ozogamicin) and ICD-derived CALR binding to NKp30 receptors (40, 41). CD25-targeted ADCs deplete immunosuppressive Tregs while preserving NK cell cytotoxicity (42). What’s more, ADCs elevate tumour-infiltrating CD8+ and Th1 cells while suppressing Treg-derived IL-10/TGF-β, thus improving the Teff/Treg ratio (42, 43). Notably, T-DXd induces epitope spreading, enabling cured mice to reject both HER2+ and HER2− tumours, indicative of antigen-agnostic immune memory (36).

### 2.4 Bidirectional regulation between ADCs and the TME

ADCs dynamically interact with the TME: (1) ADC-induced immunomodulation enhances sensitivity to subsequent therapies (17, 44); (2) Baseline TME features (e.g., tumour-infiltrating lymphocytes [TILs] density, IFN-γ signalling) predict ADC efficacy (12). For example, advanced triple-negative breast cancer (TNBC) patients with PD-1^+^ TIL-rich microenvironments exhibit superior responses to antibody-drug conjugate and ADC-ICI combinations (45). Thus it can be seen that the assessment of tumour immunogenicity within the microenvironment may provide critical guidance for ADC-based therapeutic strategies. It is also exemplified in pancreatic ductal adenocarcinoma (PDAC), where spatial heterogeneity analysis reveals that high-immunogenicity tumours (HI-PDAC) require T cell reinvigoration, whereas low-immunogenicity tumours (LI-PDAC) necessitate ICD-mediated antigen release (46). With this in mind, similar analytical methods may also enable stratification of patients according to targetable immune profiles, thereby informing precision treatment selection.

## 3 Combination therapies based on the immunomodulatory effects of ADCs

### 3.1 ADC-ICI therapies

Recent advances in combining ADCs with ICIs have demonstrated promising therapeutic potential across multiple malignancies. ADCs exert antitumour activity through targeted cytotoxic payload delivery while concurrently ICD, which promotes tumour antigen release and dendritic cell activation. This immunomodulatory mechanism provides a rational basis for synergy with ICIs, though clinical outcomes exhibit notable heterogeneity depending on tumour types and combination strategies.

#### 3.1.1 Urothelial carcinoma

In urothelial carcinoma, the phase III EV-302 trial (N=886) (47) established enfortumab vedotin (Nectin-4-targeting ADC with MMAE payload) plus pembrolizumab as a new frontline standard, showing superior progression-free survival (median PFS 12.5 vs. 6.3 months; HR=0.45, 95% CI 0.38–0.54) and overall survival (median OS 31.5 vs. 16.1 months; HR=0.47, 95% CI 0.38–0.58) compared to platinum-based chemotherapy. Despite grade ≥3 treatment-related adverse events (TRAEs) occurring in 55.9% of patients, severe toxicities like interstitial lung disease (ILD) remained rare (<1%). In contrast, a Chinese phase II study (N=16) (48) of disitamab vedotin (HER2-targeting ADC with MMAE) combined with tislelizumab reported an objective response rate (ORR) of 62.5% in pretreated patients, with HER2-positive subgroups achieving 70% ORR. While these results highlight HER2 expression as a potential predictive biomarker, small sample sizes and lack of control arms necessitate further validation.

#### 3.1.2 Breast cancer

Breast cancer research reveals divergent outcomes depending on payload characteristics. The phase Ib DS8201-A-U105 trial (N=82) (49) demonstrated trastuzumab deruxtecan (HER2-targeting ADC with topoisomerase I inhibitor deruxtecan [DXd]) plus nivolumab achieved ORRs of 65.6% in HER2-positive and 50% in HER2-low metastatic breast cancer. Preclinical evidence (38) suggests DXd upregulates PD-L1 and MHC-I expression, potentially enhancing T-cell recognition. However, a 20.7% incidence of ILD warrants stringent monitoring. Conversely, the phase II KATE2 trial (N=202) of trastuzumab emtansine (DM1 payload) with atezolizumab (50) showed no significant PFS improvement (median 8.2 vs. 6.8 months; HR=0.82, p=0.33), though PD-L1-positive subgroups trended towards benefit (HR=0.60), underscoring how payload immunomodulatory properties may influence therapeutic synergy. In addition, a trial of sacituzumab Govitecan combined with Pembrolizumab in patients with advanced breast cancer (N=104) (51) is ongoing, with a higher median PFS in the ADC-ICI arm than in the monotherapy arm in the preliminary results analysis.

#### 3.1.3 Hodgkin lymphoma

In classical Hodgkin lymphoma, brentuximab vedotin (CD30-targeting MMAE ADC) combined with nivolumab post-autologous haematopoietic stem cell transplantation yielded exceptional 18-month PFS rates of 94% (N=59) (44). Mechanistically, MMAE-induced CD30+ tumour apoptosis may enhance antigen presentation and PD-1 inhibitor-mediated immune memory. Nevertheless, peripheral neuropathy (53%) and neutropenia (42%) highlight cumulative toxicity concerns with microtubule-disrupting payloads.

#### 3.1.4 Gastric cancer

Gastric cancer studies illustrate the expanding potential of pan-HER2 strategies. A phase I trial (N=56) (17) of disitamab vedotin plus toripalimab demonstrated 50% ORR in HER2-expressing gastric/gastroesophageal junction cancer at the recommended dose, with activity maintained in HER2-low subgroups. Preclinical models further revealed complete tumour eradication and durable immune memory upon rechallenge when combining HER2-targeted ADCs with PD-1 blockade (52). Real-world data (N=38) (53) corroborated clinical efficacy (63.2% ORR, median PFS 8.2 months) without grade ≥3 TRAEs.

Despite these advances, key challenges persist. Efficacy heterogeneity across tumour types—exemplified by enfortumab vedotin’s success in urothelial carcinoma (18, 47, 54) versus trastuzumab emtansine’s limited impact in breast cancer (50)—may reflect differences in payload-mediated immunogenic potential. Furthermore, biomarker development remains inadequate, as most trials lack stratification by PD-L1 status or tumour immune microenvironment profiles. The I-SPY2.2 (55) trial’s observation of 72% pathological complete response rates in HER2-negative/immune-activated breast cancer subtypes hints at microenvironment-driven predictive factors. Information on the above researches can be found in (**Table 1**).

**Table 1:** Selected ADC-ICI clinical trials with completed or preliminary results.

PMID	NCT	ADC	Immune Checkpoint Inhibitor	Patient Population (Tumour Type/Stage)	Clinical Trial Phase	Primary Endpoints	Study Status
PMID: 39405343	NCT03523572	Trastuzumab Deruxtecan	Nivolumab	HER2+ metastatic breast cancer (mBC) and metastatic urothelial cancer (mUC)	PhaseI	Confirmed ORR: 65.6% (Cohort 1, HER2+ mBC)	Completed
PMID: 33002436	NCT02924883	Trastuzumab Emtansine	Atezolizumab	HER2+ advanced breast cancer (post-trastuzumab/taxane therapy)	PhaseII	Median PFS: 8.2 vs. 6.8 months (HR=0.82, p=0.33)	Completed
Ongoing	NCT04448886	Sacituzumab Govitecan	Pembrolizumab	Metastatic HR+/HER2- breast cancer, failure of ≥f line of endocrine therapy, 0–1 line of chemotherapy	PhaseII	Median PFS: 8.4 vs. 6.2 months (HR=0.76, p=0.26)	Ongoing
Ongoing	NCT01042379	Datopotamab Deruxtecan	Durvalumab	Neoadjuvant breast cancer	PhaseII	Pathological complete remission rate (pCR)	Ongoing
PMID: 38446675	NCT04223856	Enfortumab Vedotin	Pembrolizumab	Untreated locally advanced/metastatic urothelial carcinoma	PhaseIII	Median PFS: 12.5 vs. 6.3 months (HR=0.45); Median OS: 31.5 vs. 16.1 months (HR=0.47)	Completed
PMID: 36041086	NCT03288545	Enfortumab Vedotin	Pembrolizumab	Cisplatin-ineligible untreated locally advanced/metastatic urothelial cancer	PhaseI/II	Safety	Phase III trial ongoing: NCT04223856
PMID: 37935113	Real world	Disitamab Vedotin	PD-1 inhibitors (e.g., Toripalimab)	Locally advanced/metastatic urothelial carcinoma (pretreated)	Real-world study	ORR: 63.2% (95% CI 47.1-79.2); Median PFS: 8.2 months	Completed
PMID: 38455962	Retro	Disitamab Vedotin	Tislelizumab	Advanced urothelial carcinoma (chemotherapy-refractory)	PhaseII	ORR: 62.5%; DCR: 87.5%	Completed
PMID: 37369081	NCT03288545	Enfortumab Vedotin	Pembrolizumab	Cisplatin-ineligible untreated locally advanced/metastatic urothelial cancer	PhaseI/II	Confirmed ORR: 64.5% (combination) vs. 45.2% (monotherapy)	Completed
PMID: 38235421	NCT04280341	Disitamab Vedotin	Toripalimab	HER2-expressing advanced gastric/GEJ cancer and other solid tumours	PhaseI	Safety determination (RC48 2.5 mg/kg + Toripalimab 3 mg/kg, q2w)	Completed
PMID: 33827139	NCT02572167	Brentuximab Vedotin	Nivolumab	Relapsed/refractory classical Hodgkin lymphoma (cHL)	PhaseI/II	ORR: 85% (CR: 67%); 3-year PFS: 77%	Completed
PMID: 36403579	NCT03057795	Brentuximab Vedotin	Nivolumab	High-risk relapsed/refractory classic Hodgkin lymphoma (post-autologous HSCT)	PhaseII	18-month PFS: 94% (95% CI 84-98)	Completed

ORR, Objective Response Rate; PFS, Progression-Free Survival; CR, Complete Response; DCR, Disease Control Rate; HR, Hazard Ratio; mBC, metastatic Breast Cancer; GEJ, Gastroesophageal Junction; RC48, Disitamab vedotin (RC48-ADC, a HER2-targeted antibody-drug conjugate); NCT, National Clinical Trial identifier; PMID, PubMed Identifier; ADC, Antibody-Drug Conjugate.

**Figure 2:**
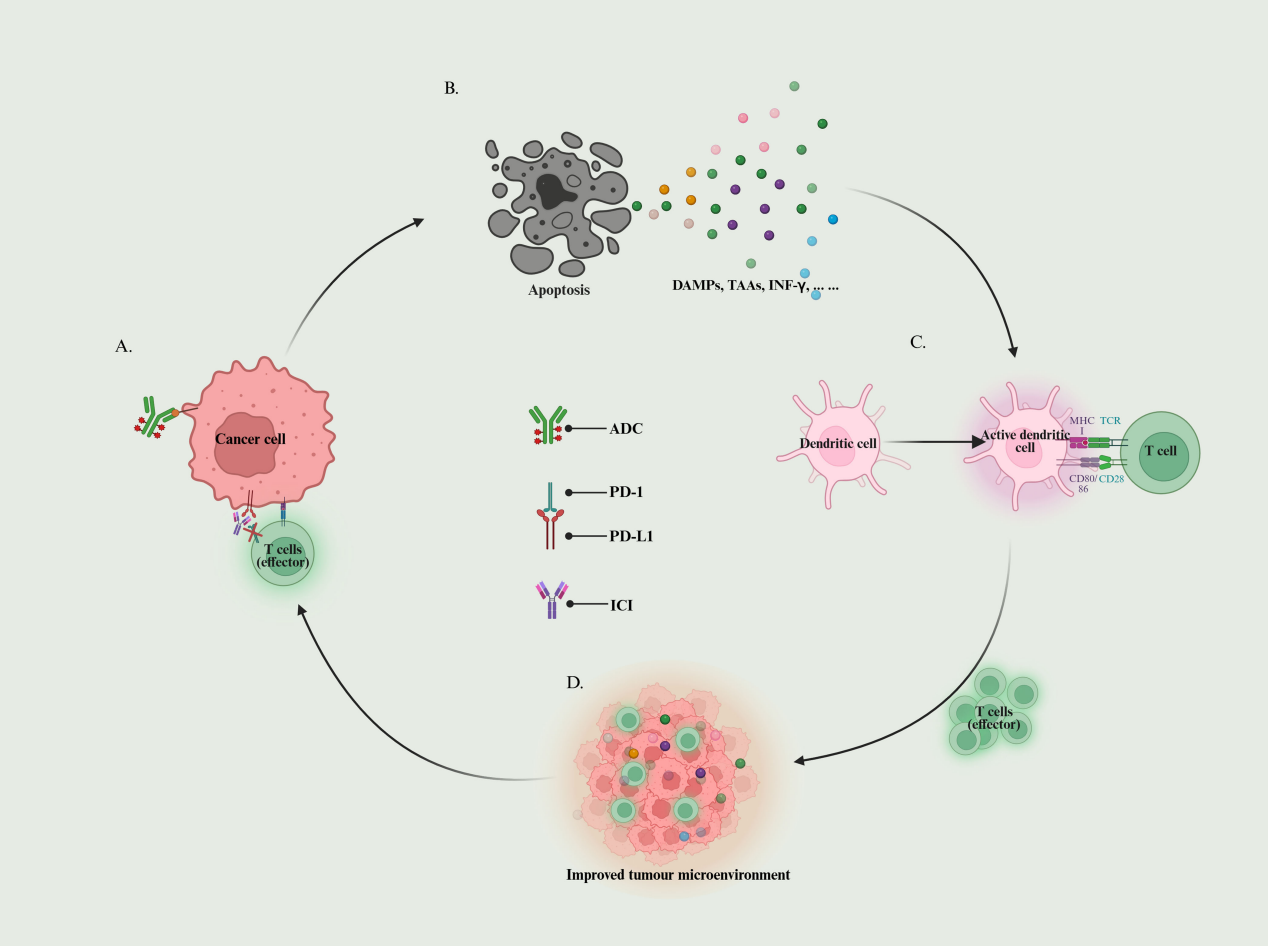
ADC-ICI synergistic cycle: **(A)** ADC recognises target antigens and ICI antagonises immune checkpoints; **(B)** Tumour cells release DAMPs, TAAs, IFN-γ, etc. to promote the activation and infiltration of immune cells; **(C)** Dendritic cells recognise TAAs and activate T cells; **(D)** Activated T cells migrate into tumour tissues to reduce the immune-suppressive state of the tumour microenvironment, and to enhance the sensitivity to ICI.

ADC-ICI combinations leverage mechanistic synergies to remodel immunosuppressive tumour microenvironments (**Figure 2**). However, clinical translation requires resolving biomarker deficiencies, managing toxicity management, and optimising temporal optimisation. Future directions should prioritise three axes: optimised sequencing strategies to balance efficacy and toxicity, novel ADC designs incorporating immune-priming payloads, and multidimensional biomarker profiling to identify patient subsets most likely to benefit from combinatorial approaches.

### 3.2 Combination strategies of ADCs with other immune-based therapies

ADCs reshape antitumour immunity by triggering immunogenic tumour cell apoptosis and enhancing cytotoxic T lymphocyte infiltration which primes the microenvironment for synergistic engagement with immunotherapies (12, 56). Beyond checkpoint blockade ADC-mediated tumour antigen modulation facilitates adoptive cell therapies as shown by polatuzumab vedotin enabling chimeric antigen receptor T-cell therapy (CAR-T) bridging in refractory lymphomas via CD79b-directed payload delivery (57). Emerging platforms integrate innate immune activation through TLR7 (Toll-like receptor 7)-agonist ISACs (Immune-Stimulating Antibody Conjugates) that conditionally stimulate myeloid cells upon tumour recognition (58) while surface-engineered NK cells conjugated with hydrophobic ADCs enable spatially controlled dual chemo-immunotherapy (59). Building on existing evidence, antibody-drug conjugates (ADCs) may synergise with cell-engineered immunotherapies (e.g., CAR-T) through target antigen upregulation. ADCs potentially enhance pro-inflammatory cytokine release and tumour-associated antigen presentation (56, 60). Additionally, they may promote infiltration of immune effector cells (such as TILs and NK cells) into the tumour microenvironment. These mechanisms collectively support ADC-immunotherapy combinations, though precise operational dynamics require further mechanistic validation through dedicated studies. Accordingly, ADCs may be combined with multiple immunotherapies to enhance therapeutic outcomes by ameliorating the tumour immune microenvironment. Future advances require temporal optimisation of combination schedules mechanistic biomarker identification and tumour-agnostic evaluation of ADC-immunotherapy partnerships to address heterogeneous resistance mechanisms.

## 4 Challenges and future perspectives

Multiple ongoing clinical trials are evaluating ADC-ICI combinations across cancer types. Despite encouraging early-phase data, these combinations confront multifaceted translational barriers. First, the predominance of phase I/II trials limits access to long-term survival data (e.g., overall survival [OS] and progression-free survival [PFS]), precluding definitive conclusions on sustained benefits. Second, combination therapy may amplify toxicity risks (e.g., immune-related adverse events and ADCs off-target effects). While some trials focus on target-specific biomarkers (e.g., HER2 or TROP2 expression), the absence of predictive biomarkers hinders patient stratification, particularly in malignancies with tumour heterogeneity. Logistical complexities (e.g., dosing sequence and timing) further complicate clinical implementation. Critically, slow trial progression (with many studies in early recruitment or planning phases) restricts data availability, impeding rapid therapeutic development. (**Table 2**)

**Table 2:** ADC-ICI clinical trials registered on ClinicalTrials.gov (ongoing or recruiting).

NCT	Study Status	Tumour Types	ADC	ADC Target	ICI	Phase	Study Type
NCT03310957	Completed	Triple Negative Breast Neoplasms	Ladiratuzumab vedotin	LIV-1	Pembrolizumab	PhaseI/II	Interventional
NCT04873362	Active	Breast Cancer	Trastuzumab emtansine	HER2	Atezolizumab	PhaseIII	Interventional
NCT05382286	Active	Triple Negative Breast Cancer, PD-L1 Positive	Sacituzumab govitecan	TROP2	Pembrolizumab	PhaseIII	Interventional
NCT05633654	Recruiting	Triple Negative Breast Cancer	Sacituzumab govitecan	TROP2	Pembrolizumab	PhaseIII	Interventional
NCT05675579	Recruiting	Breast Cancer	Sacituzumab govitecan	TROP2	Pembrolizumab	PhaseII	Interventional
NCT06081244	Recruiting	Triple Negative Breast Cancer	Sacituzumab govitecan	TROP2	Pembrolizumab	PhaseII	Interventional
NCT06899126	Not Yet Recruiting	Non-Small Cell Lung Cancer	Trastuzumab deruxtecan	HER2	Pembrolizumab	PhaseIII	Interventional
NCT06055465	Recruiting	Lung Cancer	Sacituzumab govitecan	TROP2	Pembrolizumab	PhaseII	Interventional
NCT05633667	Recruiting	Lung Cancer, Advanced or Metastatic Non-Small-Cell Lung Cancer, Resectable Non-Small-Cell Lung Cancer	Sacituzumab govitecan	TROP2	Zimberelimab, Domvanalimab	PhaseII	Interventional
NCT06764875	Recruiting	HER2-positive Gastric Cancer, Gastroesophageal Junction Adenocarcinoma	Trastuzumab deruxtecan	HER2	Rilvegostomig,	PhaseIII	Interventional
NCT06731478	Not Yet Recruiting	Gastric Cancer, Gastroesophageal Junction Cancer	Trastuzumab deruxtecan	HER2	Pembrolizumab	PhaseIII	Interventional
NCT05480384	Recruiting	Esophageal Adenocarcinoma | Esophageal Cancer, HER-2 Protein Overexpression, Gastroesophageal-junction Cancer	Trastuzumab deruxtecan	HER2	Nivolumab	PhaseII	Interventional
NCT05911295	Recruiting	Urothelial Carcinoma	Disitamab vedotin	HER2	Pembrolizumab	PhaseIII	Interventional
NCT04879329	Recruiting	Urothelial Carcinoma	Disitamab vedotin	HER2	Pembrolizumab	PhaseII	Interventional
NCT04863885	Active	Metastatic Urothelial Carcinoma	Sacituzumab govitecan	TROP2	Ipilimumab, Nivolumab	PhaseI/II	Interventional
NCT05845450	Recruiting	Colorectal Cancer, Resectable Colorectal Carcinoma	Trastuzumab deruxtecan	HER2	Durvalumab, Panitumumab	PhaseII	Interventional
NCT06682728	Recruiting	Urothelial Carcinoma, Muscle-invasive Bladder Cancer	Sacituzumab govitecan	TROP2	Nivolumab	PhaseII	Interventional
NCT06161532	Recruiting	Small Cell Carcinoma of the Bladder | Small Cell Carcinoma of the Urinary Tract, Squamous Cell Carcinoma of the Bladder, Squamous Cell Carcinoma of the Urinary Tract, Primary Adenocarcinoma of the Bladder, Primary Adenocarcinoma of the Urinary Tract, Renal Medullary Carcinoma, Squamous Cell Carcinoma of the Penis	Sacituzumab govitecan	TROP2	Atezolizumab	PhaseII	Interventional
NCT04160494	Active	Malignant Glioma	D2C7-IT	EGFRwt & EGFRvIII	Atezolizumab	PhaseI	Interventional
NCT03835819	Active	Endometrial Cancer	IMGN853	FRα	Pembrolizumab	PhaseII	Interventional
NCT06563778	Not Yet Recruiting	Ineligible Or Refused Transplant Patients With Classical Hodgkin Lymphoma	Brentuximab vedotin	CD30	Anti-PD-1 antibody	PhaseII	Interventional
NCT06043674	Recruiting	Chronic Lymphocytic Leukemia, Richter’s Transformation	Polatuzumab vedotin	CD79b	Glofitamab, Obinutuzumab	PhaseII	Interventional
NCT05039073	Recruiting	Recurrent Classic Hodgkin Lymphoma, Refractory Classic Hodgkin Lymphoma	Brentuximab vedotin	CD30	Nivolumab	PhaseII	Interventional

ADC, Antibody-Drug Conjugate; ICI, Immune Checkpoint Inhibitor; NCT, National Clinical Trial identifier; HER2, Human Epidermal Growth Factor Receptor 2; TROP2, Trophoblast Cell Surface Antigen 2; CD30, Cluster of Differentiation 30; CD79b, Cluster of Differentiation 79b; EGFRwt, Epidermal Growth Factor Receptor wild type; EGFRvIII, Epidermal Growth Factor Receptor variant III; FRα, Folate Receptor Alpha; LIV-1, Solute Carrier Family 39 Member 6.

Despite the remarkable anti-tumour potential of ADCs through ICD and TME remodelling, their clinical translation faces critical challenges. The immunomodulatory potential of contemporary antibody-drug conjugates remains insufficiently harnessed. First, the lack of validated biomarkers limits precision-patient stratification. Current markers like PD-L1 expression and TIL density inadequately reflect TME heterogeneity. For example, spatial immunosuppressive gradients in pancreatic cancer compromise single-biopsy assessments (46), while dynamic TME changes — such as IFN-γ signalling fluctuations—further obscure response prediction. Standardisation of ICD-related biomarkers (e.g., HMGB1, ATP) across payload classes is also urgently needed.

Toxicity management remains a major hurdle for ADC-ICI combinations. Overlapping adverse events, notably ILD and neurotoxicity, require proactive mitigation. Topoisomerase inhibitor-based ADCs (e.g., trastuzumab deruxtecan) combined with PD-1 inhibitors exhibit ILD rates up to 20% (49), while microtubule-targeting payloads (e.g., MMAE) exacerbate peripheral neuropathy, often necessitating dose reductions. Resistance mechanisms further complicate outcomes: antigen heterogeneity limits bystander effects (e.g., T-DM1’s inefficacy in HER2-low tumours due to non-cleavable linkers), while ADC-induced PD-L1 upregulation may accelerate adaptive immune evasion (61).

Novel payload research continues to target the remodelling of the immunosuppressive tumour microenvironment (39, 62). The microbially inspired CD47-listeriolysin O (LLO) conjugate exemplifies this untapped capacity by employing mechanism (63): disruption of phagocytic checkpoints coupled with lysosomal escape to activate the cGAS–STING pathway and strengthen tumour antigen cross-presentation, thereby remodelling TME immunogenicity. However, clinical translation of such novel ADCs necessitates an equilibrium between immunostimulatory intensity and drug safety. Furthermore, explorations of other strategies — including the double antibody-drug conjugate (DAD) (64) and the dual-payload antibody-drug conjugate (65, 66) — could also circumvent the limitations of traditional cytotoxicity-centric directions. Future progress depends on the engineering and mechanistic synergy exploration of ADCs.

## 5 Conclusion

In conclusion, the immunomodulatory mechanism of ADCs play an important part in the anti-tumour immune response. Partial researches confirm that ADCs may have the potential of synergy with ICIs. However, the effect of combination therapy demands resolution of biomarker, safety, and resistance challenges to complete a multifaceted assessment. In addition, the immune regulatory mechanism of ADCs also needs to be clarified at a more profound level to provide theoretical basis for novel research focuses.
